# Outcomes and Quality of Life Improvement After Multilevel Spinal Fusion in Elderly Patients

**DOI:** 10.1177/2192568219849393

**Published:** 2019-05-19

**Authors:** John M. Ibrahim, Paramjit Singh, Daniel Beckerman, Serena S. Hu, Bobby Tay, Vedat Deviren, Shane Burch, Sigurd H. Berven

**Affiliations:** 1 University of California–San Francisco, San Francisco, CA, USA; 2 Stanford University Medical Center, Palo Alto, CA, USA

**Keywords:** deformity, fusion, quality of life, elderly, predictor, outcome

## Abstract

**Study Design:**

Retrospective case series.

**Objectives:**

Both the rate and complexity of spine surgeries in elderly patients has increased. This study reports the outcomes of multilevel spine fusion in elderly patients and provides evidence on the appropriateness of complex surgery in elderly patients.

**Methods:**

We identified 101 patients older than70 years who had ≥5 levels of fusion. Demographic, medical, and surgical data, and change between preoperative and >500 days postoperative health survey scores were collected. Health surveys were visual analogue scale (VAS), EuroQoL 5 Dimensions (EQ-5D), Oswestry Disability Index (ODI), Scoliosis Research Society questionnaire (SRS-30), and Short Form health survey (SF-12) (physical composite score [PCS] and mental composite score [MCS]). Minimal clinically important differences (MCIDs) were defined for each survey.

**Results:**

Complications included dural tears (19%), intensive care unit admission (48%), revision surgery within 2 to 5 years (24%), and death within 2 to 5 years (16%). The percentage of patients who reported an improvement in health-related quality of life (HRQOL) of at least an MCID was: VAS Back 69%; EQ-5D 41%; ODI 58%; SRS-30 45%; SF-12 PCS 44%; and SF-12 MCS 48%. Improvement after a primary surgery, as compared with a revision, was on average 13 points higher in ODI (*P* = .007). Patients who developed a surgical complication averaged an improvement 11 points lower on ODI (*P* = .042). Patients were more likely to find improvement in their health if they had a lower American Society of Anesthesiologists or Charlson Comorbidity Index score or a higher metabolic equivalent score.

**Conclusions:**

In multilevel surgery in patients older than 70 years, complications are common, and on average 77% of patients attain some improvement, with 51% reaching an MCID. Physiological status is a stronger predictor of outcomes than chronological age.

## Introduction

Spinal disorders are common and present a significant burden on affected patients and the health care economy. Spinal pathology is especially prevalent among the elderly, including degenerative pathology, spinal deformity, tumors, and fractures. With aging of the US population, the burden of spinal disorders on health status and health care expenditure will continue to increase.^1^ In 2010, the US Census Bureau estimated the number of people aged 65 years or older at 40.5 million, or 13% of the total US population, which is projected to double by 2050.^2^ Prevalence estimates indicate that adult spinal deformity affects approximately 27.5 million elderly patients, which will likely be closer to 60 million patients in 2050.^3^ In this growing, elderly population, the appropriate management of spinal deformity is not well-defined.^4^

In the past decade, there has been a significant increase in both the rate and complexity of spine surgeries for elderly patients.^5,6^ These patients account for a disproportionate share of health care expenditures, and the cost of complex spinal surgeries is increasing.^7^ The number of >3 level spinal fusions in patients older than 60 years increased from 6571 in 2004 to 16 526 in 2011, and average hospital charges increased from $90 096 to $187 230 during this same time period.^7^ The observed increase in the rate, complexity, and cost of spinal surgery in older patients drives the priority of studying the outcomes of surgery and providing evidence to guide appropriate care.

There is significant variability between and within spine centers regarding the management of spinal disorders in elderly patients.^8^ Determining whether surgery is appropriate has become increasingly relevant. Appropriate surgery, as defined by the RAND/UCLA Appropriateness Method, is one where the expected benefits of a procedure outweigh the expected risks.^9^ The potential benefits of spine surgery include improvement in pain, discomfort, function, and quality of life.^10^ Potential risks include increased pain, neural injury, infection, need for revision surgery and death.^11,12^ Information on expected outcomes, including risk and benefits, is important for guiding informed choice of patients and physicians. The purpose of this study to is to report the outcome of multilevel spine fusion in elderly patients and to provide evidence to guide decision making regarding the appropriateness of complex surgery in elderly patients.

## Methods

### Design

This was a retrospective case series of elderly patients treated with multilevel spinal fusion.

### Cohort

**Figure 1.**
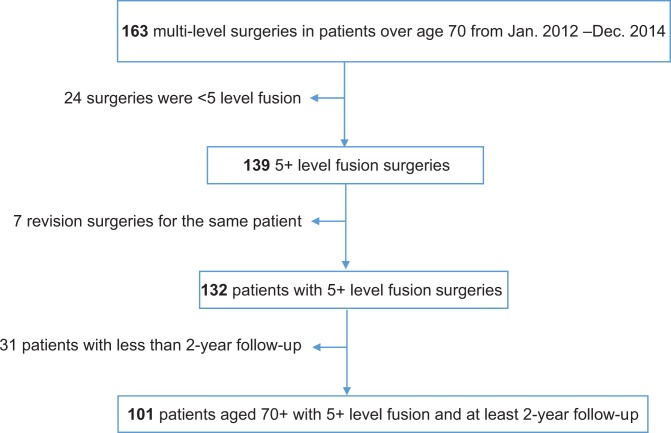
Eligibility and cohort selection.

Approval was obtained from the Human Research Protection Program (HRPP) Institutional Review Board at the university (study number 16-21 339). Patients who had undergone a multi-level spinal operation were identified using a university hospital database. Health information from patients at the university’s spine center was collected prospectively by 5 surgeons and their assistants into a central database. Database search criteria were patients 70 years old or older and who had undergone surgery between January 2012 and December 2014. Eligible operations were those with an instrumented fusion of 5 or more levels. Patients were excluded if 2-year follow-up data was unavailable (Figure 1).

### Data Collection

Predictor variables included age, gender, body mass index (BMI), multiple comorbidities including osteoporosis (DEXA *T*-score < −2.5), American Society of Anesthesiologists (ASA) score, Charlson Comorbidity Index (CCI), metabolic equivalents (METs), surgical indication and diagnosis, whether the operation was a primary or revision surgery, surgical procedure performed, proximal and distal levels of fusion, and preoperative health-related quality of life (HRQOL) as measured by visual analogue scale (VAS), EuroQoL 5 dimensions (EQ-5D), Oswestry Disability Index (ODI), Scoliosis Research Society questionnaire (SRS-30), and Short Form health survey (SF-12) (physical composite score [PCS] and mental composite score [MCS]). Outcome variables included perioperative complications, estimated blood loss (EBL), admission to the intensive care unit (ICU), hospital length of stay (LOS), discharge status, readmissions, reoperations, death, and HRQOL at least 2 years postoperatively as measured by VAS, EQ-5D, ODI, SRS-30, and SF-12 (PCS and MCS).

Each HRQOL survey measures a different aspect of health. VAS measures pain and ranges from 0 (no pain) to 10 (maximum pain). EQ-5D measures general health status in five dimensions: mobility, self-care, usual activities, pain/discomfort, and anxiety/depression and ranges from 0 (death or no health) to 1 (perfect health). ODI quantifies disability resulting from low-back pain and ranges from 0 (no disability) to 100 (maximum disability). The SRS-30 measures health in 5 domains: function/activity, pain, self-image/appearance, mental health, and satisfaction with management. Scores range from 1 (worst outcome) to 5 (best outcome). The 12-item SF-12 measures 8 components of general health: physical functioning, role limitations due to physical health, pain, general health, vitality, social function, role limitations due to emotional problems, and mental health. The SF-12 has 2 composite scores, PCS and MCS, and ranges from 0 (lowest level of health) to 100 (highest level of health).

### Statistical Analysis

Improvement in HRQOL was determined absolutely, and relative to the minimal clinically important differences (MCIDs) reported in the literature for each survey. The MCID for each survey was as follows: VAS score difference of 2; EQ-5D score difference of 0.15; ODI score difference of 10; SRS-30 score difference of 0.4; and SF-12 score difference of 5 for both PCS and MCS.^13,14^

Independent *t* test and Pearson’s correlation coefficients were calculated for the association between (1) achieving an MCID and (2) dichotomous and continuous variables, respectively. Univariate logistic regression was performed to determine predictors of worse postoperative quality of life. Variables with a *P* value of less than .10 on univariate analysis were included in a multivariate logistic regression model. Statistical significance was set at *P* = .05.

## Results

A total of 101 patients were included in our final analysis (Table 1). The majority were female (73%) and the average age was 74.9 years (range 70-88 years). The mean BMI was 27.8 ± 5.8 kg/m^2^, ASA score was 2.4 ± 0.5, CCI was 1.1 ± 1.4, and METs was 4.6 ± 1.4. A documented diagnosis of osteoporosis was present in 19% of patients and a diagnosis of adult spinal deformity was present in 53% of patients. The average number of levels fused was 9.4 ± 3.5, and 56% of all surgeries were revision surgeries.

**Table 1.** Patient Characteristics.

Characteristic	Value (N = 101)
Age, years mean (range)	74.9 (70-88)
Female, no	73
BMI, kg/m^2^, mean ± SD	27.8 ± 5.8
ASA, mean ± SD	2.4 ± 0.5
CCI, mean ± SD	1.1 ± 1.4
METs, mean ± SD	4.6 ± 1.4
Diagnosis: Adult spinal deformity, %	53
Primary surgeries, %	44
Levels fused, mean ± SD	9.4 ± 3.5

Abbreviations: BMI, body mass index; ASA, American Society of Anesthesiologists; CCI, Charlson Comorbidity Index; MET, metabolic equivalent.

The perioperative course is summarized in Table 2. Intraoperatively, average EBL was 1258 ± 908 mL and dural tears occurred in 19% of patients. Overall, 69% of dural tears occurred in patients undergoing revision surgery. Medical complications occurred in 56% of patients and surgical complications in 15%. Common perioperative events included blood loss anemia requiring transfusion (58%), admission to the ICU (48%), an additional revision surgery within 2 to 5 years (24%), and death within 2 to 5 years (16%). Average hospital length of stay was 8.1 ± 3.3 days and patients were most likely to be discharged to an acute rehab facility (43%) or skilled nursing facility (17%).

**Table 2.** Perioperative Course.

EBL, mL, mean ± SD	1258 ± 908
Dural tears, %	19
Admission to ICU, %	48
Need for transfusion, %	58
Medical complication, %	56
Surgical complication, %	15
Length of stay, days, mean ± SD	8.1 ± 3.3
Discharge status, %	
Acute rehabilitation	43
SNF	17
Home with home PT/OT/nursing	16
Home	16
Hospital	1
Revision surgery within 2-5 years, %	24
Death within 2-5 years, %	16

Abbreviations: EBL, estimated blood loss; ICU, intensive care unit; SNF, skilled nursing facility; PT, physical therapy; OT, occupational therapy.

**Figure 2.**
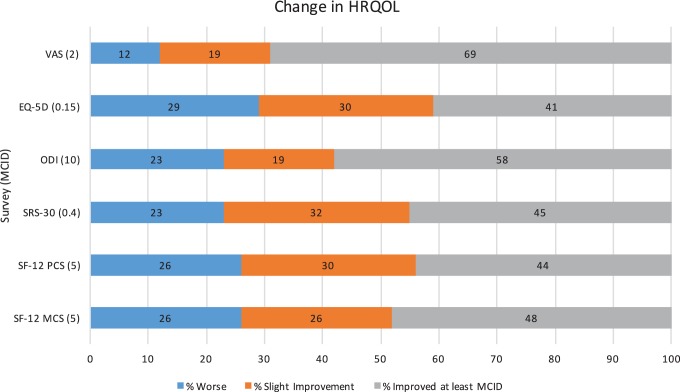
Change between preoperative and 2-year postoperative quality of life. Percentages of patients who improved at least an MCID (in gray), who had slight improvement (in orange), and who were worse (in blue) at 2-years postoperatively are displayed.

The average improvement in health status from preoperative to at least 2 years after surgery reached the MCID threshold in all surveys. Average improvement for VAS Back was 2.3 ± 3.7, EQ-5D was 0.18 ± 0.23, ODI was 13.6 ± 16.4, SRS was 0.44 ± 0.55; SF-12 PCS was 5.8 ± 15.9; and SF-12 MCS was 6.5 ± 14.8 (Table 3). Improvement in at least 1 survey was reported in 91% of patients. The percentage of patients who reported an improvement in HRQOL of at least an MCID was: VAS Back 69%; EQ-5D 41%; ODI 58%; SRS-30 45%; SF-12 PCS 44%; and SF-12 MCS 48% (Figure 2).

**Table 3.** Average Improvements in Each HRQOL Survey.

Survey (MCID)	Mean Improvement
VAS (2)	2.3 ± 3.7
EQ-5D (0.15)	0.18 ± 0.23
ODI (10)	13.6 ± 16.4
SRS-30 (0.4)	0.44 ± 0.55
SF-12 PCS (5)	5.8 ± 15.9
SF-12 MCS (5)	6.5 ± 14.8

Abbreviations: HRQOL, health-related quality of life; MCID, minimum clinically important difference; VAS, visual analogue scale; EQ-5D, EuroQoL 5 Dimensions; ODI, Oswestry Disability Index; SRS-30, Scoliosis Research Society questionnaire; SF-12, 12-item Short Form health survey; PCS, physical composite score; MCS, mental composite score.

Table 4 shows the impact of predictors on HRQOL score improvement. Improvement after a primary surgery, as compared to a revision, was on average 13 points higher in ODI (*P* = .007). In contrast, patients who developed a surgical complication averaged an improvement 11 points lower on ODI (*P* = .042). Patients who were more active preoperatively (higher METs) had greater improvement in EQ-5D (*r* = 0.367, *P* = .033) and SF-12 PCS (*r* = 0.588, *P* = .045). Both increasing BMI (*r* = −0.44, *P* = .047) and increasing EBL (*r* = −0.487, *P* = .025) were negatively correlated with improvements in SF-12 MCS. Longer length of stay was correlated with greater score improvement in EQ-5D at final follow-up (*r* = 0.290, *P* = .031).

**Table 4.** Impact of Predictor Variables on Score Improvement.

	Difference in Improvement	Correlation	*P*
EQ-5D			
METs		0.367	.033
Length of stay		0.29	.031
ODI			
Primary surgery	13.13		.007
Surgical complication	−11.49		.042
SF-12 PCS			
METs		0.588	.045
SF-12 MCS			
BMI		−0.44	.047
EBL		−0.487	.025

Abbreviations: EQ-5D, EuroQoL 5 Dimensions; MET, metabolic equivalent; ODI, Oswestry Disability Index; SF-12, 12-item Short Form health survey; PCS, physical composite score; MCS, mental composite score; BMI, bosy mass index; EBL, estimated blood loss.

In all, 38% of patients self-reported a decline in health status in at least 1 domain. Table 5 shows the predictors of reporting a worse postoperative quality of life score, compared with preoperative health. Higher ASA was associated with worse EQ-5D (odds ratio [OR] 16, *P* = .012) and worse SF-12 PCS (OR 11.25, *P* = .041). Higher CCI (OR 1.92, *P* = .032) was associated with worse EQ-5D scores. In contrast, higher METs were protective against worse EQ-5D (OR 0.49, *P* = .022). Discharge to home with home health (OR 21, *P* = .014) and to home (OR 16.8, *P* = .021) were both associated with worse ODI scores. Cardiac complications during the perioperative course were associated with worse SRS-30 scores (OR 28, *P* = .038). Having a future revision surgery was associated with worse VAS (OR 7.4, *P* = .011) and SF-12 PCS and MCS (OR 24, *P* = .021). After multivariate logistic regression, variables that retained statistically significant associations were having a future revision surgery with worse VAS and discharge to home with home health or discharge home with worse ODI.

**Table 5.** Predictors of Reporting a Worse Postoperative Health-Related Quality of Life.^a^

	Univariate Logistic Regression			Multivariate Logistic Regression		
	Odds Ratio	95% CI	*P*	Odds Ratio	95% CI	*P*
VAS						
Length of stay	0.72	030-1.04	.083	0.65	043-1.00	.052
Future revision surgery required	**7.4**	**1.57-34.94**	**.011**	**10.57**	**1.84-60.69**	**.008**
EQ-5D						
ASA	**16**	**1.83-140.03**	**.012**	12.34	0.81-188.62	.071
CCI	**1.92**	**1.06-3.49**	**.032**	1.62	0.63-4.16	.32
METs	**0.49**	**0.27-0.90**	**.022**	0.43	0.18-1.03	.059
ODI						
Primary	0.16	0.02-1.36	.093	0.18	0.02-1.97	.16
Discharge status (ref Acute rehabilitation)						
Skilled nursing facility	3	0.16-54.57	.458	1.95	0.10-37.09	.656
Home with home health	**21**	**1.83-240.52**	**.014**	**2043**	**1.58-264.83**	**.021**
Home	**16.8**	**1.53-184.92**	**.021**	**16.07**	**1.35-191.02**	**.028**
SRS						
Age	1.34	0.95-1.90	.095	1.49	0.68-3.24	.315
Perioperative cardiac complication	**28**	**1.21-648.81**	**.038**	—	—	.997
Perioperative pulmonary complication	13	0.77-219.11	.075	—	—	.997
SF-12 PCS						
Future revision surgery required	**24**	**1.62-356.64**	**.021**	—	—	.984
Surgical complication	10.67	0.72-158.50	.086	—	—	.992
ASA	**11.25**	**1.11-114.37**	**.041**	—	—	.992
SF-12 MCS						
Future revision surgery required	**24**	**1.62-356.64**	**.021**	—	—	.996
EBL	1.01	1.00-1.01	.056	—	—	.996
Perioperative infection	10.67	0.72-158.50	.086	—	—	.996
Surgical complication	10.67	0.72-158.50	.086	—	—	.996

Abbreviations: VAS, visual analogue scale; EQ-5D, EuroQoL 5 Dimensions; ASA, American Society of Anesthesiologists; CCI, Charlson Comorbidity Index; MET, metabolic equivalent; SRS, Scoliosis Research Society; SF-12, 12-item Short Form health survey; PCS, physical composite score; MCS, mental composite score; EBL, estimated blood loss. ^a^Bolded values represent predictors with *P* < .05.

## Discussion

Multilevel fusion for spinal disorders in patients older than 70 years are common and present an important issue for our health care economy. Our study demonstrates variability in outcomes and complications for this cohort. Complications commonly occur during and following surgery, and many patients report a meaningful improvement in health. Patients were more likely to find improvement in their health if they had a higher preoperative health and functional capacity as measured by ASA, CCI, and METs, if they were undergoing a primary surgery, if they were discharged to either an acute rehabilitation or skilled nursing facility, and if they did not require a future revision surgery.

Clinical outcomes were variable in this cohort. Across all surveys, an average of 51% of patients reached an MCID, 26% either remained the same or had slight improvement, and 23% reported a worse health status. Although complication rates were high, almost all patients (91%) had improvement in at least one of the HRQOL surveys from baseline. In a study by Smith et al^15^ on the risks and benefits of adult scoliosis surgery, patients over the age of 65 had greater baseline disability and pain, higher perioperative complication rates, but greater improvement in disability and pain after surgery when compared with younger patients. Interestingly, increasing hospital length of stay was correlated with improvement in EQ-5D. One possible explanation is that patients with longer hospital stays undergo more complex procedures and have a lower preoperative EQ-5D and consequently have greater improvement from surgery. This notion of greater potential for improvement being related to more severe disease and more complicated surgeries may also explain why patients discharged to an acute rehabilitation or skilled nursing facility report greater improvements.

On average, estimated intraoperative blood loss was greater than 1 L and over half of patients received a transfusion for blood loss anemia, similar values as those in prior studies.^16,17^ Just under half of patients were admitted to the ICU and a quarter had revision surgery within 2 to 5 years of surgery. We found that 16% of our cohort had died within 2 to 5 years after their surgery. In the United States, life expectancy for males and females at age 70 years is 15.6 years, and the probability of dying between ages 70 and 75 years is 11%, and between ages 75 and 80 is 17%.^18^ The average age of our cohort was 75 years, so our reported death rate is similar to the national death rate. Complication rates were consistent with other reports in the literature.^19,20^

It is known that preoperative BMI is a significant predictor of complications after spine surgery.^21^ Preoperative optimization, specifically having a BMI <35 kg/m^2^ and better preoperative mobility, is associated with reduced complications.^22^ Likewise, this study identified that physiologic parameters such as BMI, ASA, CCI, and METs are important predictors of HRQOL improvement. Additionally, we found patients undergoing a primary spine surgery reported greater improvement in their health compared with those undergoing revision surgery, likely because revisions are technically more challenging and complications are more common.^23,24^ Furthermore, patients that required a future revision surgery were more likely to be worse off, suggesting that primary surgeries represent an opportunity to provide the maximum benefit for the patient. Age did not have a significant impact on reported health, implying that chronological age may be less important than physiological characteristics in predicting outcomes after spine surgery. Additional research is needed to clarify the impact of inpatient perioperative management on health for this cohort of patients. This emphasizes the need for a holistic preoperative evaluation in conjunction with this data. Predictive modeling will play an instrumental role in better identifying specific preoperative predictors of good and bad outcomes.

Physicians should carefully consider if nonoperative treatments or more limited surgeries are appropriate alternative treatments, while considering risks of revision surgery for such limited interventions. For elderly patients without a progressive neural deficit or spinal instability, initial non-operative treatment is appropriate.^25^ These include physical therapy, pain medications, and injections.^26^ Operative treatment of adult deformity is appropriate in patients with a progressive neural deficit, progressive deformity, or for pain or functional limitations after nonoperative options have been exhausted.^25^ Depending on patient symptoms, operative treatments include decompression, decompression with limited arthrodesis, and complex realignment of the spine.^25^ Some patients may be candidates for minimally invasive surgeries (MIS) and the minimally invasive spinal deformity surgery (MISDEF) algorithm can help in selection.^27^ Predictive modeling to identify patients who are most likely to benefit from nonoperative or more limited operative intervention is an important next step.

The main strength of this article is that we report granular predictor and outcome data—including long-term postoperative quality of life in several domains—for a specific patient population and procedure where limited other data exists. Our work differs from previous studies as our outcome variables include 5 common QOL surveys used by spine surgeons. This makes it a comprehensive report on the impact of preoperative demographic, medical, and surgical predictors on overall postoperative quality of life, rather than only focusing on a single aspect of postoperative health. This study demonstrates a level of specificity that will better guide informed choice in selecting a treatment to address a patient’s chief complaint given his or her unique characteristics. Through this work, we intend to inspire future studies of predictive modeling to further identify strong predictors of outcomes. Nevertheless, this study has limitations. First, although this data was collected prospectively, this study was designed as a retrospective study. Second, we determined improvement in health via an MCID value, which is an imperfect measure of improvement. For example, an improvement in VAS from 10 to 7 is not the same as from 3 to 0, yet both meet the MCID criteria for VAS. It does, however, provide a consistent, convenient measure for tracking meaningful change. Last, including several HRQOL surveys makes it difficult to identify common predictors of reported improvements as each survey measure a different aspect of health, limiting the strength of multivariate regression models.

## Conclusion

Appropriate surgery is surgery in which the benefits of the intervention exceed the risk. In multilevel surgery in patients older than 70 years, complications are common, and on average 77% of patients attain some improvement, with 51% reaching an MCID. Physiological status is a stronger predictor of outcomes than chronological age. Patients undergoing revision surgery or those who develop a surgical complication are more likely to have lower improvement. Knowledge of observed outcomes including risks and benefits of surgery will empower informed choice regarding management of spinal disorders in elderly patients.
